# Anticancer Effect of Second-line Treatment for Castration-Resistant Prostate Cancer Following First-line Treatment with Androgen Receptor Pathway Inhibitors

**DOI:** 10.31662/jmaj.2021-0163

**Published:** 2021-12-28

**Authors:** Takashi Matsumoto, Masaki Shiota, Shigetomo Yamada, Leandro Blas, Hidekazu Naganuma, Ken Lee, Keisuke Monji, Eiji Kashiwagi, Ario Takeuchi, Junichi Inokuchi, Ken-ichiro Shiga, Akira Yokomizo, Masatoshi Eto

**Affiliations:** 1 Department of Urology, Graduate School of Medical Sciences, Kyushu University, Fukuoka, Japan; 2 Department of Urology, Harasanshin Hospital, Fukuoka, Japan

**Keywords:** docetaxel, castration-resistant prostate cancer, androgen receptor pathway inhibitor, Radium-223

## Abstract

**Introduction:**

Studies on the effect of androgen receptor pathway inhibitors (ARPI), docetaxel (DTX), and radium-223 (Ra-223) after first-line treatment with ARPI in patients with castration-resistant prostate cancer (CRPC) are scarce. This study compared the efficacy of treatment after ARPI for CRPC.

**Methods:**

Patients with CRPC who received ARPI as first-line treatment and different second-line treatments were retrospectively reviewed. Clinicopathological backgrounds and treatment outcomes, including maximum prostate-specific antigen (PSA) decrease, progression-free survival (PFS), and overall survival (OS), were compared between second-line treatments.

**Results:**

In total, 88 patients were enrolled. Forty-one (46.6%), 37 (42.0%), and 10 (11.4%) patients were treated with ARPI, DTX, and Ra-223, respectively. Patients whose PSA levels were not adequately reduced by first-line treatment with ARPI were eventually enrolled in the DTX treatment (P = 0.030). PSA decrease was not significantly different when comparing treatments. PFS in the DTX group was significantly better than in the other two groups (P = 0.023). In multivariate analysis, DTX was an independent prognostic factor for better PFS compared to ARPI (hazard ratio, 95% confidence interval; 0.44, 0.25-0.79, P = 0.006). Subgroup analysis showed a favorable impact of DTX on PFS in patients with Gleason score >8 (interaction P = 0.027) and a PSA decline >50% (interaction P = 0.019) during first-line treatment with ARPI. However, no significant difference in OS was observed between groups of different second-line treatments.

**Conclusions:**

This study suggests that in patients with CRPC, second-line treatment with DTX following progression in patients who received ARPI as first-line treatment is more beneficial compared with second-line treatment with ARPI or Ra-233.

## Introduction

In 2004, a study demonstrating that docetaxel (DTX) prolonged survival in patients with metastatic castration-resistant prostate cancer (CRPC) ^(1), (2)^ made a breakthrough in the treatment of this condition ^(3), (4)^. Later, androgen receptor pathway inhibitors (ARPI) were added to the treatment armamentarium. A CYP17 inhibitor (abiraterone) and a second-generation antiandrogen (enzalutamide) improved survival in patients with chemotherapy-naïve metastatic CRPC ^(5), (6)^. With time, first-line ARPI following androgen-deprivation therapy has become a preferred therapeutic option for CRPC ^(7)^. Radium-223 dichloride (radium-223) and DTX after first-line treatment with ARPI have shown benefits for bone-metastatic CRPC ^(8)^. However, the optimal treatment sequence after first-line ARPI remains unclear.

In retrospective studies, it has been reported that alternating two ARPIs (abiraterone and enzalutamide) offered limited efficacy ^(9), (10)^. In addition, Chi et al. have conducted a phase II clinical trial using both ARPI (abiraterone and enzalutamide), and the efficacy of alternating therapies of both ARPIs was not significant ^(11)^. In the PLATO study, patients received enzalutamide and at PSA progression were assigned to receive abiraterone plus placebo or abiraterone plus enzalutamide; the efficacy of second-line therapy was limited, with a PSA decline >50% of 1%-2% ^(12)^. Clinical trials comparing the efficacy of different second-line treatment after first-line ARPI for CRPC have not been conducted. Here we investigated the therapeutic outcomes of second-line treatments following first-line treatment with ARPI in patients with CRPC.

## Materials and Methods

### Patients

We included patients treated with a second-line agent after first-line treatment with ARPI for CRPC between 2014 and 2018. Eligibility criteria included (i) histopathologically diagnosed carcinoma of the prostate, (ii) confirmed failure of first-line treatment with ARPI, and (iii) age ≥20 years. Clinical staging was determined using the uniform TNM criteria based on the results of digital rectal examination, transrectal ultrasound, magnetic resonance imaging, computed tomography, and bone scan ^(13)^. All patients underwent needle biopsy regardless of radical prostatectomy, and biopsy Gleason score was utilized in this study. The extent of disease score was divided into five grades according to the degree of bone metastasis, as shown by scan as follows ^(14)^: 0, normal; 1, less than six bone metastases, each being ≤50% of size of vertebral body (one lesion with the size of vertebral body accounted as two lesions); 2, 6-20 bone metastases; 3, >20 bone metastases but less than a “super scan”; and 4, “super scan” or bone metastases involving >75% of ribs, vertebrae, and pelvic bones. Baseline clinical characteristics and serum data were obtained retrospectively from the patients’ medical records. Written informed consent was obtained from all patients. This study (# 2019-230) was performed in accordance with the principles described in the Declaration of Helsinki and the Ethical Guidelines for Epidemiological Research enacted by the Japanese Government and approved by the institutional review board of Kyushu University and Harasanshin Hospital.

### Exposure

All patients had received abiraterone or enzalutamide as first-line treatment until the disease progression, as defined by the Prostate Cancer Working Group criteria ^(15)^. After confirmed failure, patients received an ARPI, DTX, or Ra-233 as second-line treatment. An ARPI including abiraterone (1,000 mg/day) with prednisolone (10 mg/day) or enzalutamide (160 mg/day) was administered as previously described ^(16), (17)^. DTX (70-75 mg/m^2^) was administered every 3 or 4 weeks as reported elsewhere ^(18), (19)^. Ra-223 was administered every 4 weeks according to the standard treatment regimen ^(8)^. Castration status by surgical or continuous medical castration with a luteinizing hormone-releasing hormone agonist (goserelin acetate or leuprorelin acetate) or antagonist (degarelix acetate) was maintained simultaneously during treatment. Doses and schedules were modified according to the severity of adverse events in each case. Treatment was discontinued according to the physician’s discretion, based on disease progression, adverse events, or patient’s refusal.

### Endpoints

Disease progression was defined as (i) an increase in serum prostate-specific antigen (PSA) of >2 ng/ml, (ii) a 50% increase over the nadir, and/or (iii) the appearance of a new lesion or progression of one or more known lesions classified according to the Response Evaluation Criteria in Solid Tumors version 1.1 ^(15)^. The primary outcome of this analysis was progression-free survival (PFS) during second-line treatment with ARPI, DTX, or Ra-233. PFS and overall survival (OS) were calculated from the starting date of second-line treatment to the date of disease progression in the case of PFS and death from any cause in the case of OS. Surviving patients without disease progression or mortality were censored at the last follow-up visit.

### Statistical analysis

All statistical analyses were performed using EZR version 1.50 software (Jichi Medical University Saitama Medical Center, Saitama, Japan) ^(20)^. Comparison between the three groups was performed using the Kruskal-Wallis test. Survival was estimated using the Kaplan-Meier method, and the log-rank test was used to compare groups. Univariate and multivariate analyses were performed using the Cox proportional hazards regression model. We estimated the impact on survival of DTX under subgroup analysis according to age (<75 or ≥75 years), Gleason score (≤8 or >8), bone metastasis, visceral metastasis, time to CRPC (≥12 or <12 months), first-line agent, maximum PSA decrease during first-line treatment (≤50% or >50%), and median PSA at second-line treatment (<30 or ≥30 ng/ml). Differences in the prognostic impact of subgroups were investigated through interaction tests. The propensity score, that is, the probability of survival, was calculated using a logistic regression model in which potential confounders were as follows: age, Gleason score, bone metastasis, visceral metastasis, time to CRPC, first-line agent, maximum PSA decrease during first-line treatment, and median PSA at second-line treatment. One-to-one propensity score-matched pairs were selected from the two groups by nearest match. All tests were two-sided, and a P < 0.05 was considered statistically significant.

## Results

### Clinical characteristics

A total of 88 Japanese patients were included in this study. Table 1 lists the clinical characteristics. Forty-one (46.6%), 37 (42.0%), and 10 (11.4%) patients were treated with an ARPI, DTX, and Ra-223, respectively. Median follow-up was 10.0 months (interquartile range, 5.0-21.9 months). Patients that received ARPI were older; the median age in the groups that received ARPI, DTX, and Ra-223 were 76, 74, and 73 years, respectively (P = 0.015). There was no significant difference in biopsy Gleason score at diagnosis. Fifteen (36.6%), 14 (37.8%), and 3 (30.0%) patients received previous local therapy in ARPI, DTX, and Ra-223 group, respectively. Metastasis to the lymph node (P = 0.86), bone (P = 0.77), and viscera (P = 0.37) was comparable between groups with ARPI, DTX, and Ra-223. No significant difference in median time to treatment failure was observed in first-line treatment (P = 0.32) or in median PSA before initiating second-line treatment (P = 0.10). Intriguingly, maximum PSA decrease during first-line treatment was significantly different between the three groups (P = 0.030); PSA decrease in first-line ARPI was lower in patients treated with DTX.

**Table 1.** Clinical Characteristic between ARPI, DTX, and Ra-223 as Second-line Treatment.

Variables	ARPI (n = 41)	DTX (n = 37)	Ra-223 (n = 10)	P-value
Median age, yrs [IQR]	76 [72-83]	74 [66-78]	73 [70-75]	0.015*
Median PSA at diagnosis, ng/ml [IQR]	40.6 [19.3-201.3]	86.5 [22.8-375.0]	56.9 [13.5-166.4]	0.68
Gleason score, n [%]				
≤8	19 [47.5]	12 [33.3]	2 [20.0]	0.20
>8	21 [52.5]	24 [66.7]	8 [80.0]
Previous radical local treatment, n [%]				
Absence	26 [63.4]	23 [62.2]	7 [70.0]	0.90
Presence	15 [36.6]	14 [37.8]	3 [30.0]
Median time to CRPC, mo [IQR]	19.8 [8.8, 45.7]	16.1 [9.5-27.8]	18.2 [12.0-25.4]	0.76
Lymph node metastasis at first-line treatment, n [%]				
Absence	30 [73.2]	25 [67.6]	7 [70.0]	0.86
Presence	11 [26.8]	12 [32.4]	3 [30.0]
Bone metastasis at first-line treatment, n [%]				
Absence	13 [31.7]	11 [29.7]	2 [20.0]	0.77
Presence	28 [68.3]	26 [70.3]	8 [80.0]
Visceral metastasis at first-line treatment, n [%]				
Absence	40 [97.6]	34 [91.9]	10 [100.0]	0.37
Presence	1 [2.4]	3 [8.1]	0 [0.0]
First-line treatment agent, n [%]				
Abiraterone	17 [41.5]	12 [32.4]	3 [30.0]	0.64
Enzalutamide	24 [58.5]	25 [67.6]	7 [70.0]
Median PSA at first-line treatment, ng/ml [IQR]	16.0 [7.0-49.7]	14.2 [5.1-36.5]	4.2 [3.0-9.7]	0.046*
Median time to treatment failure in first-line treatment, mo [IQR]	7.2 [3.6-14.7]	8.0 [3.1-24.5]	17.3 [8.4-23.7]	0.32
Median of maximum PSA decrease in first-line treatment, % [IQR]	−73.5 [−93.4- -41.3]	−43.8 [−82.2, 39.7]	−85.9 [−89.0, −57.3]	0.030*
Median PSA at second-line treatment, ng/ml [IQR]	24.9 [6.1-77.4]	39.0 [11.7-200.7]	8.9 [1.9-28.7]	0.10

*statistically significant. ARPI, androgen receptor pathway inhibitor; DTX, docetaxel; Ra-223, radium-223; IQR, interquartile range; PSA, prostate-specific antigen; CRPC, castration-resistant prostate cancer

### Maximum PSA decrease during second-line treatment

**Figure 1.**
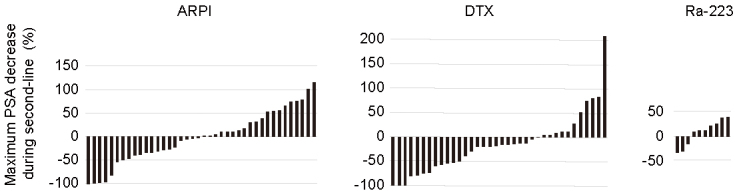
Waterfall plot of the maximum decline in PSA during second-line treatment for patients with castration-resistant prostate cancer treated with androgen receptor pathway inhibitor (ARPI), docetaxel (DTX), and radium-223 (Ra-223).

Figure 1 shows waterfall plots of maximum PSA decrease during second-line treatment. Data were missing for two patients, one patient treated with ARPI and the other, with DTX. PSA decrease was unavailable in one patient in the ARPI group. PSA decline was observed in 50% (20/40), 66.7% (24/36), and 30.0% (3/10) of patients treated with ARPI, DTX, and Ra-233, respectively (P = 0.051) (Figure 1). In particular, a PSA decline of >50% was observed in 15% (6/40), 33.3% (12/36), and 0% (0/10) patients with ARPI, DTX, and Ra-223, respectively, with a statistically significant difference between groups (P = 0.033). Of note, none of the patients treated with Ra-223 had a decline of PSA >50%.

### Prognosis in second-line treatment

**Figure 2.**
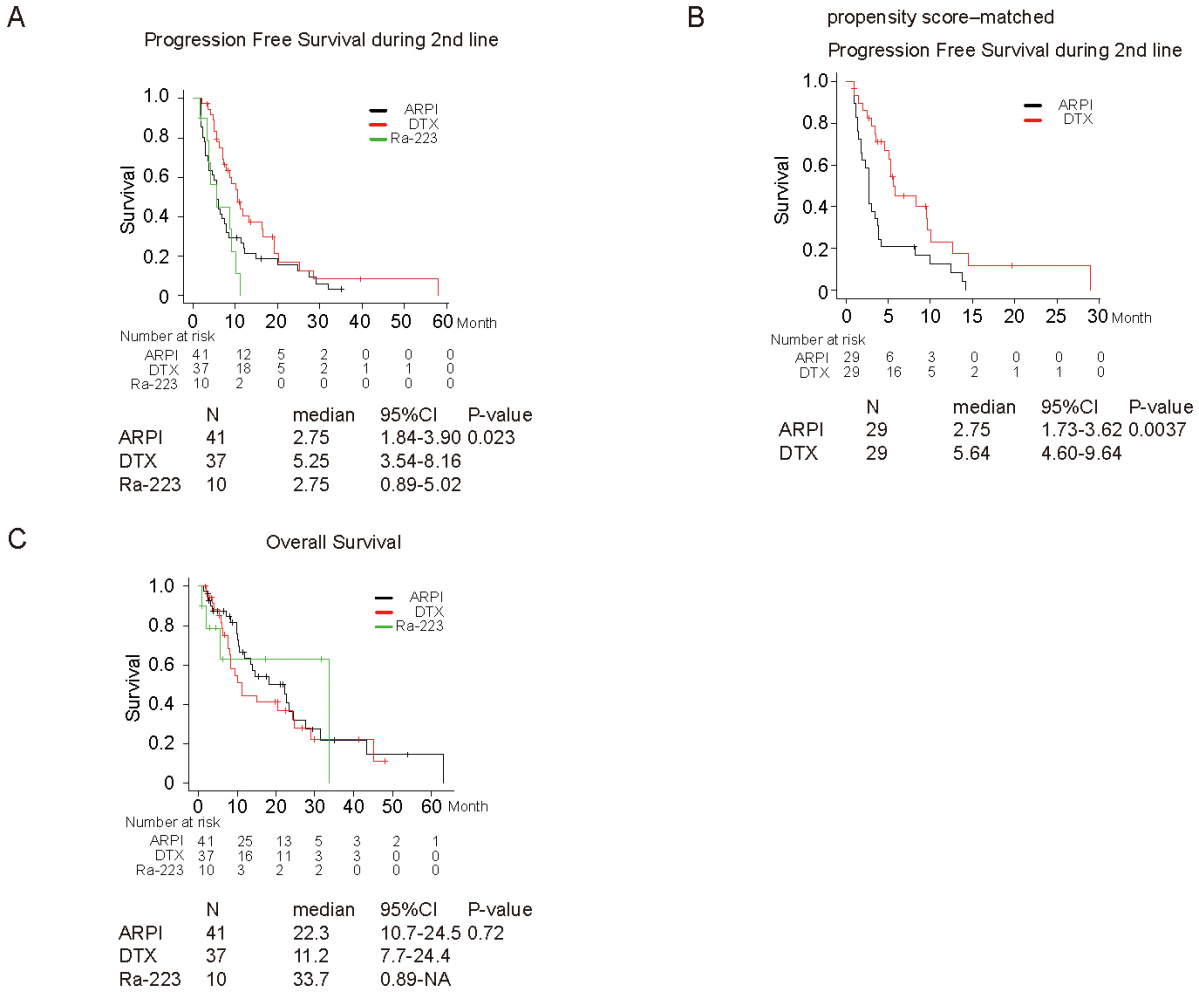
Kaplan-Meier survival curve for patients treated with androgen receptor pathway inhibitor (ARPI), docetaxel (DTX), and radium-223 (Ra-223) as second-line treatment in terms of progression-free survival (A), propensity score-matched progression-free survival (B), and overall survival (C).

Figure 2 shows PFS and OS in ARPI, DTX, and Ra-223 groups. PFS during second-line treatment was significantly better in DTX (median, 95% confidence interval [CI]; 5.3 months, 3.5-8.2 months) compared to those in ARPI (median, 95% CI; 2.8 months, 1.8-3.9 months) and Ra-223 group (median, 95% CI; 2.8 months, 0.9-5.0 months) (P = 0.023; DTX vs. ARPI, P = 0.044, DTX vs. Ra-223, P = 0.0019, ARPI vs. Ra-223, P =0.36, Figure 2A). DTX was an independent prognostic factor for better PFS compared to ARPI in univariate (hazard ratio [HR], 95% CI; 0.60, 0.37-0.99, P = 0.046) and multivariate analyses (HR, 95% CI; 0.54, 0.25-0.79, P = 0.006) (Table 2). We included 29 patients in each group using propensity score matching to compare the PFS between second-line treatments (ARPI vs. DTX). PFS was significantly better in the group with DTX (median, 95% CI; 5.6 months, 4.6-9.6 months) than in the group that received ARPI (median, 95% CI; 2.8 months, 1.7-3.6 months) (P = 0.0037; Figure 2B). No significant difference in OS was observed between groups of different second-line treatments (Figure 2C).

**Table 2.** Univariate and Multivariate Analysis on Variables Associated with PFS.

	Univariate			Multivariate		
Variables	HR	95% CI	P-value	HR	95% CI	P-value
Age
<75 years (ref)
≧75 years	1.23	0.78-1.95	0.37	1.19	0.72-1.98	0.50
Gleason score						
≤8 (ref)						
>8	1.31	0.82-2.08	0.26	1.32	0.77-2.25	0.31
Bone metastasis						
Absence (ref)						
Presence	1.35	0.79-2.31	0.28	1.01	0.56-1.81	0.99
Visceral metastasis						
Absence (ref)						
Presence	0.63	0.20-2.01	0.43	0.56	0.16-1.99	0.37
Time to CRPC						
≧12 months (ref)						
<12 months	0.89	0.55-1.43	0.62	0.66	0.38-1.14	0.14
First-line treatment agent						
Abiraterone (ref)						
Enzalutamide	0.96	0.59-1.54	0.85	1.29	0.75-2.21	0.36
Maximum PSA decrease during first-line treatment						
≤50 % (ref)						
>50 %	0.75	0.47-1.20	0.23	0.63	0.36-1.11	0.11
PSA at second-line treatment
<30 ng/ml (ref)
≧30 ng/ml	1.21	0.76-1.91	0.42	1.43	0.83-2.45	0.19
Second-line treatment
ARPI (ref)
DTX	0.60	0.37-0.99	0.046*	0.44	0.25-0.79	0.0061*
Ra-223	1.55	0.76-3.19	0.23	1.39	0.60-3.21	0.44

*statistically significant. HR, hazard ratio; CI, confidence interval; ARPI, androgen receptor pathway inhibitor; DTX, docetaxel; Ra-223, radium-223; CRPC, castration-resistant prostate cancer

**Figure 3.**
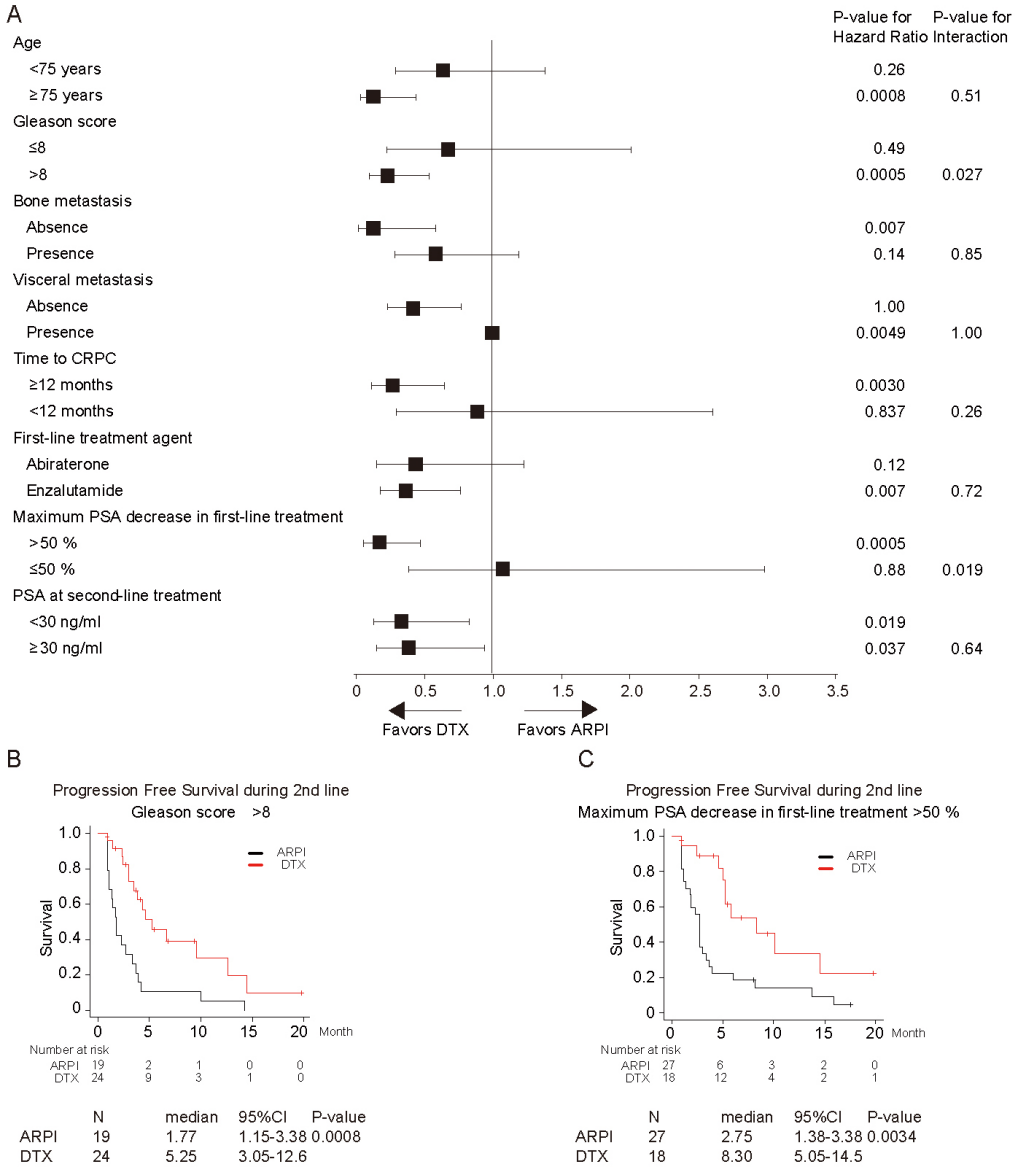
Subgroup analysis of progression-free survival for patients treated with androgen receptor pathway inhibitor (ARPI), docetaxel (DTX). The hazard rate (■) with 95% CIs is plotted in forest plot (A). P-values for hazard ration and interaction test are shown stratified by age, Gleason score, bone metastasis, visceral metastasis, time to castration-resistant prostate cancer (CRPC), first-line treatment agent, maximum PSA decrease in first-line treatment, and PSA at second-line treatment. Kaplan-Meier survival curves of progression-free survival in patients with castration-resistant prostate cancer stratified by Gleason score (> 8) (B) and maximum PSA decrease in first-line treatment (C).

Finally, subgroup analyses were performed (Figure 3A). In patients with Gleason score ≤8 vs. >8, ARPI and DTX showed a significantly different impact on PFS (interaction P = 0.027). PFS was significantly better in the group with DTX (median, 95% CI; 5.3 months, 3.1-12.6 months) than in the group that received ARPI (median, 95% CI; 1.8 months, 1.2-3.4 months) in patients with Gleason score >8 (P = 0.0008; Figure 3B). The same was observed in subgroups of patients with PSA decline >50% vs. ≤50% (interaction P = 0.019). PFS was significantly better in the group with DTX (median, 95% CI; 8.3 months, 5.1-14.5 months) than in the group that received ARPI (median, 95% CI; 2.8 months, 1.4-3.4 months) in patients with maximum PSA response >50% (P = 0.0034; Figure 3C).

## Discussion

This study suggests that second-line treatment with DTX following progression on first-line with ARPI is potentially beneficial compared with second-line ARPI in patients with CRPC. Of note, OS was comparable between the two groups.

Retrospective studies have investigated the efficacy of DTX therapy following ARPI for patients with CRPC. Miyake et al. reported that the PSA response, PFS, and OS during second-line therapy in the DTX group were significantly superior to those for the ARAT group in patients with metastatic CRPC ^(21)^. Matsubara et al. evaluated the prognosis of 139 patients with CRPC treated with alternating ARPIs or switched to DTX following first-line ARPI and showed a significantly better PFS in the group that received DTX as second-line treatment compared with ARPI ^(22)^. Similarly, Oh et al. studied 345 patients with metastatic CRPC treated with chemotherapy (DTX/CBZ) or ARPI ^(23)^. PSA response, time to PSA progression, and the objective response were better in the chemotherapy group compared with the ARPI in patients with poor prognostic features (hemoglobin < 11 g/dl, LDH > upper limit of normal, albumin < lower limit of normal). Moreover, those receiving chemotherapy had significantly improved OS. A phase III randomized controlled trial (CARD trial) showed that a novel taxane cabazitaxel chemotherapy significantly improved clinical outcomes, including PFS and OS, compared with ARPI (abiraterone or enzalutamide), in patients with metastatic CRPC who had been previously treated with DTX and ARPI ^(24)^. Taken together, these results suggest that taxane chemotherapy is an appropriate therapeutic option as a subsequent treatment for metastatic CRPC after first-line ARPI.

Subgroup analysis in this study showed a favorable impact of DTX on PFS in patients with Gleason score >8 and a PSA decline >50% during first-line treatment with ARPI. Interestingly, this study showed the importance of Gleason score at initial diagnosis even in second-line treatment for CRPC. Previous reports indicated that the presence of Gleason pattern 5, including tertiary (<5%), is a strong prognosticator in later-line settings ^(25), (26)^. This finding suggested that cancer component with Gleason pattern 5 persisted and regrew even after primary treatment, and then Gleason score at initial diagnosis is still a clinically valuable parameter in this setting. Because a high Gleason score represents poor prognosis, findings showing that DTX was more beneficial to patients with a high Gleason score seems to be consistent with the study by Oh et al., which indicated that anticancer efficacy was better in chemotherapy compared with ARPI among patients with poor prognostic features ^(22)^.

This study has several limitations. First, clinical data were collected retrospectively, and some data were missing. Second, the number of patients in each group was small, especially in Ra-223 group. Third, the clinical background of patients may be different from that of ARPI and DTX groups, since radium-223 is indicated only for bone metastasis without visceral metastasis. In addition, Ra-223 is a disadvantage in PSA response. However, radiographic progression was not evaluated during second-line treatment due to the nature retrospective study.

### Conclusion

Our findings suggest that DTX may have a superior anticancer efficacy as a second-line treatment for CRPC following first-line treatment with ARPI. Therefore, switching treatment from ARPI to chemotherapy may be an appropriate strategy.

## Article Information

### Conflicts of Interest

M.S. and A.Y. have received honoraria from Janssen Pharma, Astellas Pharma, AstraZeneca, Bayer, and Sanofi. E.M. received honoraria from Takeda and Janssen and a scholar donation from Sanofi, Astellas, Takeda, and Bayer

### Acknowledgement

The authors thank the many staffs in the Kyushu University and Harasanshin Hospital who assisted in this study.

### Author Contributions

T.M. is the first author and wrote this manuscript. M.S. designed and managed this study. A.Y. and M.E. organized this study between two hospitals for this study. L.B. proofread this manuscript. Others (S.Y., H.N., K.L., K.M., E.K., A.T., J.I., and K.S.) designed this study and collected the clinical data for this study from patient’s record.

### Approval by Institutional Review Board (IRB)

This study (# 2019-230) was approved by institutional review board of Kyushu University and Harasanshin Hospital.
